# The use of a scented face mask in pediatric patients may facilitate mask acceptance before anesthesia induction

**DOI:** 10.3389/fmed.2023.1190728

**Published:** 2023-06-02

**Authors:** Yukako Abukawa, Koji Takano, Yuko Hobo, Erika Hosaka, Ayano Kimura, Yoshifumi Suga, Nobuyuki Katori, Tsunehisa Tsubokawa

**Affiliations:** ^1^Department of Anesthesiology, Jikei University School of Medicine, Tokyo, Japan; ^2^Department of Anesthesiology, School of Medicine, Juntendo University, Tokyo, Japan

**Keywords:** scented mask, mask acceptance, pediatric, anesthesia induction, behavioral

## Abstract

**Background:**

Scented face masks are commonly used during the induction phase of anesthesia. The present study investigated whether the use of a scented mask improved mask acceptance before the slow induction of anesthesia in pediatric patients.

**Methods:**

This prospective, randomized controlled trial enrolled patients aged 2–10 years who were scheduled to undergo surgery under general anesthesia. Patients were randomly assigned to either of regular unscented (control group) or scented (experimental group) face masks before anesthesia induction with a parent. The primary outcome was the mask acceptance score, rated on a validated 4-point from 1 point (not afraid; easily accepts the mask) to 4 points (afraid of a mask; crying or struggling). The secondary outcome was heart rate assessed by pulse oximetry in the pediatric ward before transfer to the operating room (OR), at the entrance to the OR, at the patient notification of mask fitting by the anesthesiologist, and after mask fitting.

**Results:**

Seventy-seven patients were accessed for eligibility, with 67 enrolled in the study: 33 in the experimental group and 34 in the control group. Mask acceptance was significantly greater among patients aged 2–3 years in the experimental than in the control group (*p* < 0.05).

**Conclusion:**

The use of a scented mask can improve mask acceptance before anesthesia induction with a parental presence in pediatric patients aged 2–3 years.

**Clinical Trial Registration**: https://upload.umin.ac.jp/cgi-open-bin/ctr_e/ctr_view.cgi?recptno=R000040819.

## Background

Inhalation of the volatile anesthetic agent via a face mask, so-called slow induction, is generally employed for the induction of general anesthesia to reduce physical and psychological stresses in pediatric patients. Satisfactory mask acceptance is important for a smooth induction process; however, some patients experience discomfort or fear of the mask and refuse mask fitting, which can complicate successful anesthesia induction. The use of a scented mask, which can disguise the odor of the mask and/or inhaled anesthetic agents, has been used for anesthesia induction in pediatric patients, as it may improve mask acceptance (1, 2).

There have been made many efforts to facilitate the induction of general anesthesia in pediatric patients: premedication with sedative agents such as midazolam or dexmedetomidine (3), parental presence during the induction of anesthesia (4, 5), giving toys (6), video games (7), and clown doctors (8). Showing video programs to the patient during slow induction is another resort to facilitate anesthesia induction in pediatric patients; however, the effectiveness of audiovisual stimuli was dependent on the patient’s age (9). This result inspired us that the effectiveness of a scented mask to facilitate induction in pediatric patients may also be dependent on age. Few studies to date, however, have analyzed age-dependent differences in the effects of scented masks.

This study hypothesized that the use of a scented mask would facilitate the before-induction phase of general anesthesia in pediatric patients, and that mask acceptance may be age-dependent. This study, therefore, compared the acceptance of scented and unscented masks in pediatric patients categorized according to age.

## Methods

This clinical trial was registered with the University Hospital Medical Information Network Clinical Trial Registry.^1^ The study protocol was approved by the Jikei University Hospital Ethics Committee (ID number: 30–242, approved on 7/Aug/2019), and written and oral informed consent was obtained from all parents and/or patients. This single-institution prospective study was performed in accordance with the Declaration of Helsinki and Clinical Trials Act established by the Japanese Ministry of Health, Labour and Welfare.

This prospective, randomized controlled study included pediatric patients (age: 2–10 years) who were scheduled to undergo general anesthesia from August 2019 to December 2020 at the Jikei University Hospital. The data were saved to a secured external storage unit on site. Patients were included if they had an American Society of Anesthesiologists (ASA) physical status of 1 or 2. Patients with intellectual disability, developmental delay, or without a documented pulse rate (PR) were excluded, as were uncooperative patients, defined as those with difficulty wearing the pulse oximeter. Patients were stratified by age into 3 groups: group 1; patients aged 2–3, group 2; 4–6 years, and group 3; 7–10 years (9).

Patients in each age group were randomly assigned to undergo masking with a scented (strawberry, cherry, or gum) face mask (experimental group) or an unscented face mask (control group). To reduce selection bias, the envelope method was used for randomization. An anesthesiologist who did not participate in the study selected one piece of paper from the envelope for each patient. We showed all patients and their parents a mask the day before anesthesia and explained to them how we were going to place the mask on the patient’s face. The patients or their parents in the experimental group chose the flavor of the scented mask the day before surgery. All patients underwent preoperative fasting, and none received premedication. The primary outcome was the mask acceptance score. Mask acceptance, as evaluated by an independent observer or/and the attending anesthesiologist, was rated on a scale of 1 to 4 points, with 1 point indicating no fear of the mask and its easy acceptance; 2 points indicating a slight fear of the mask, with the patient being easy to comfort; 3 points indicating moderate fear of the mask, with the patient being difficult to calm; and 4 points indicating fear of the mask, with the patient crying or struggling (10). Simultaneously, the behavioral score was calculated according to the following parameters: distress behaviors, including crying, screaming, nonverbal resistance, verbal resistance, and negative verbal emotion (11). The behavioral score was evaluated based on the parameters in Table 1, which was scored between 0 and 5 points. These scorings were evaluated immediately after the mask fitting basically within 5–10 s. The secondary outcome was PR, as assessed by pulse oximetry at four time points: (1) at baseline in the pediatric ward before proceeding to the operating room (OR), (2) at the entrance to the OR, (3) at the patient notification of mask fitting by the anesthesiologist, and (4) after mask fitting. Patients were accompanied by either or both of their parents on the way from the pediatric ward to the OR. One of the parents was sitting on a chair beside the patient until the induction of anesthesia according to our clinical practice. Patients were shown the selected scented face mask (Ambu King Mask; Ambu A/S, Ballerup, Denmark) or an unscented face mask prior to placing it on the patient’s face. At this point, only oxygen was provided so that the effect of the mask itself could be assessed by an independent anesthesiologist. None of the inhalational agents was administered prior to the evaluation of the scores and pulse rate.

**Table 1:** Behavioral score.

Parameter	Score
Crying	1
Screaming	1
Nonverbal resistance: e.g., trying to remove the mask, flapping their arms and/or legs, shaking their head	1
Verbal resistance: e.g. “No,” “Take it off”	1
Negative verbal emotion: e.g. “I’m scared,”” I do not like it”	1
Total (between 0 and 5)	

Based on our preliminary study, we assumed that the percentage of patients showing a mask acceptance score of 2 or less accounted for 20 and 80% in the control and experimental groups, respectively. We calculated the appropriate sample size for each group as 10, assuming an alpha error of 5% and statistical power of 80%. Patient demographic and baseline clinical data, behavioral score, and PR are expressed as mean (SD). And the mask acceptance score is expressed as median (inter-quartile range). Statistical analysis was performed using SigmaPlot version 13 (Systat Software, Inc., San Jose, CA, United States). The normal distribution of mask acceptance scores was determined using the Bartlett test for equal variances, followed by one-way analysis of variance and the Bonferroni multiple comparison test. The normal distribution of PR was tested using the Shapiro–Wilk test followed by analysis using repeated-measures, and two-way analysis of variance. PRs in the control and experimental groups for each age category were evaluated by multiple pairwise comparisons using the Holm-Šidák method. *p* values <0.05 were considered statistically significant.

## Results

**Figure 1:**
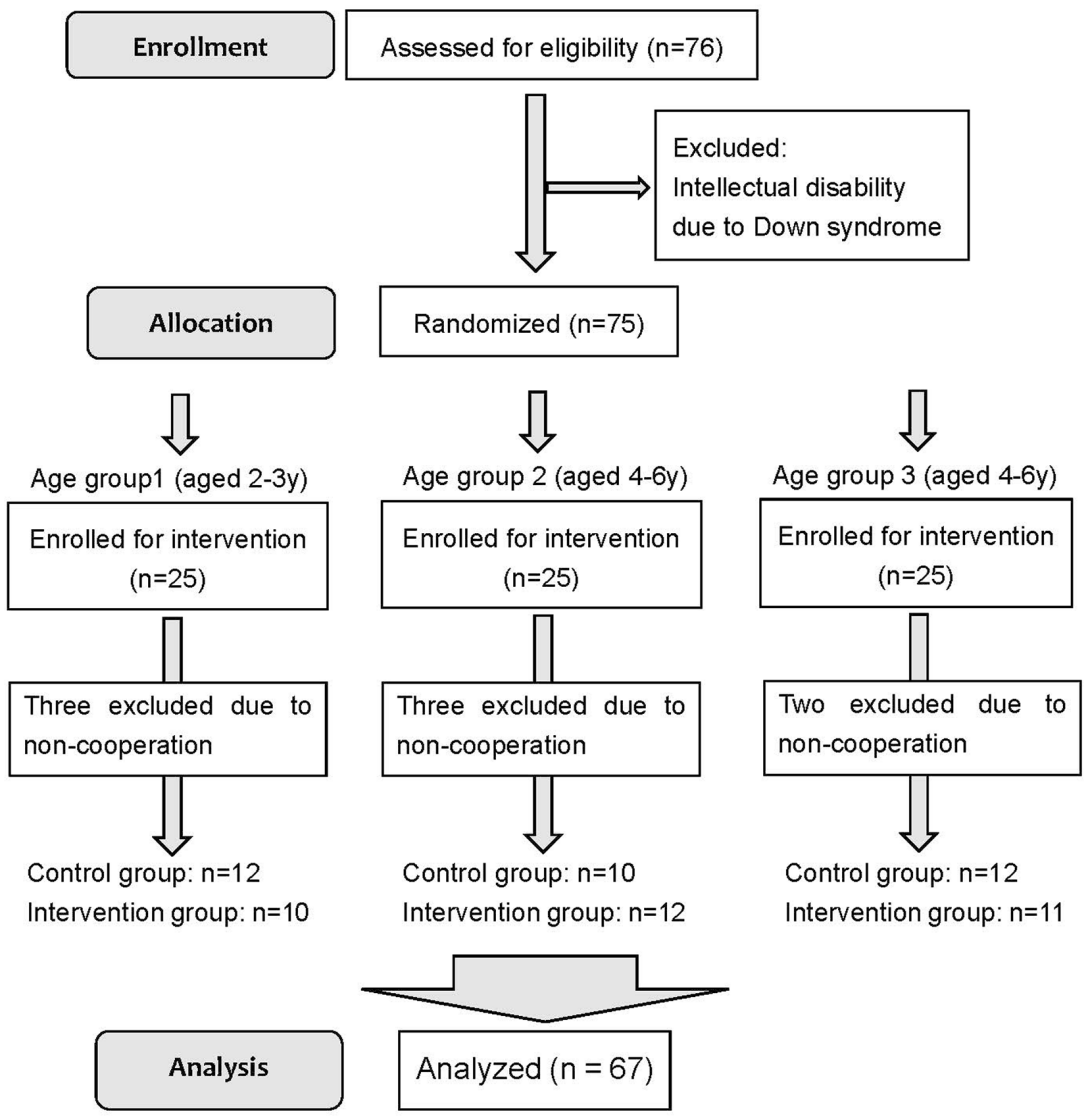
Patient flow diagram.

Figure 1 shows a Consolidated Standards of Reporting Trials (CONSORT) flow diagram for the patients in this study from August 2019. Of the 77 patients recruited, 10 were excluded, including one patient with Down syndrome, one without a parent present during anesthesia, and eight who refused to wear a pulse oximeter before entering the OR (Figure 1). The 67 patients included 33 in the experimental group and 34 in the control group. No harmful event was identified during and after the study period.

**Figure 2:**
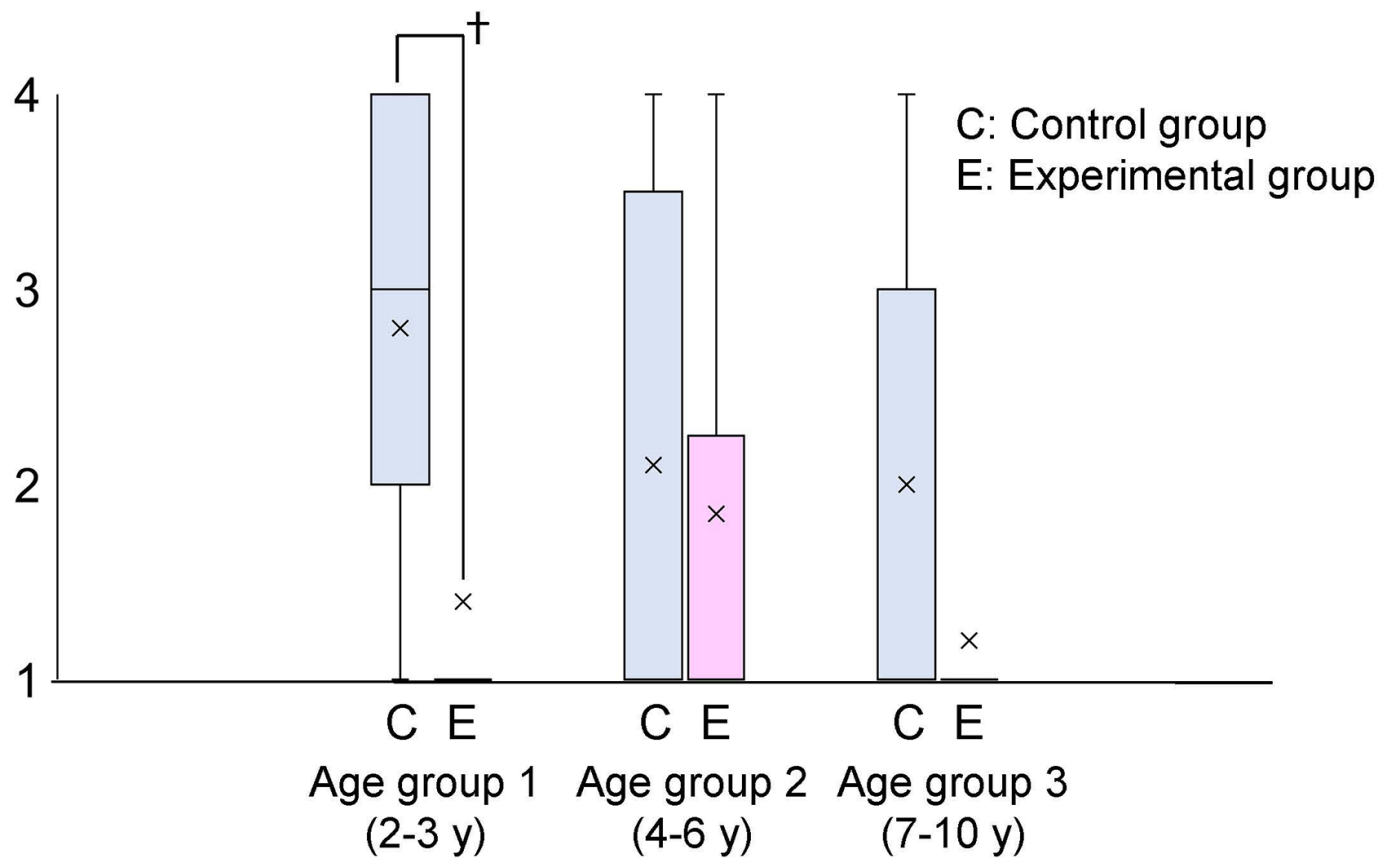
Mask acceptance score by age group C and E express control and experimental groups, respectively. The mask acceptance score was significantly lower in patients wearing a scented mask than those wearing an unscented one in age group 1 (†: *p* < 0.05). “X” in the graph expresses the mean values in each group.

**Figure 3:**
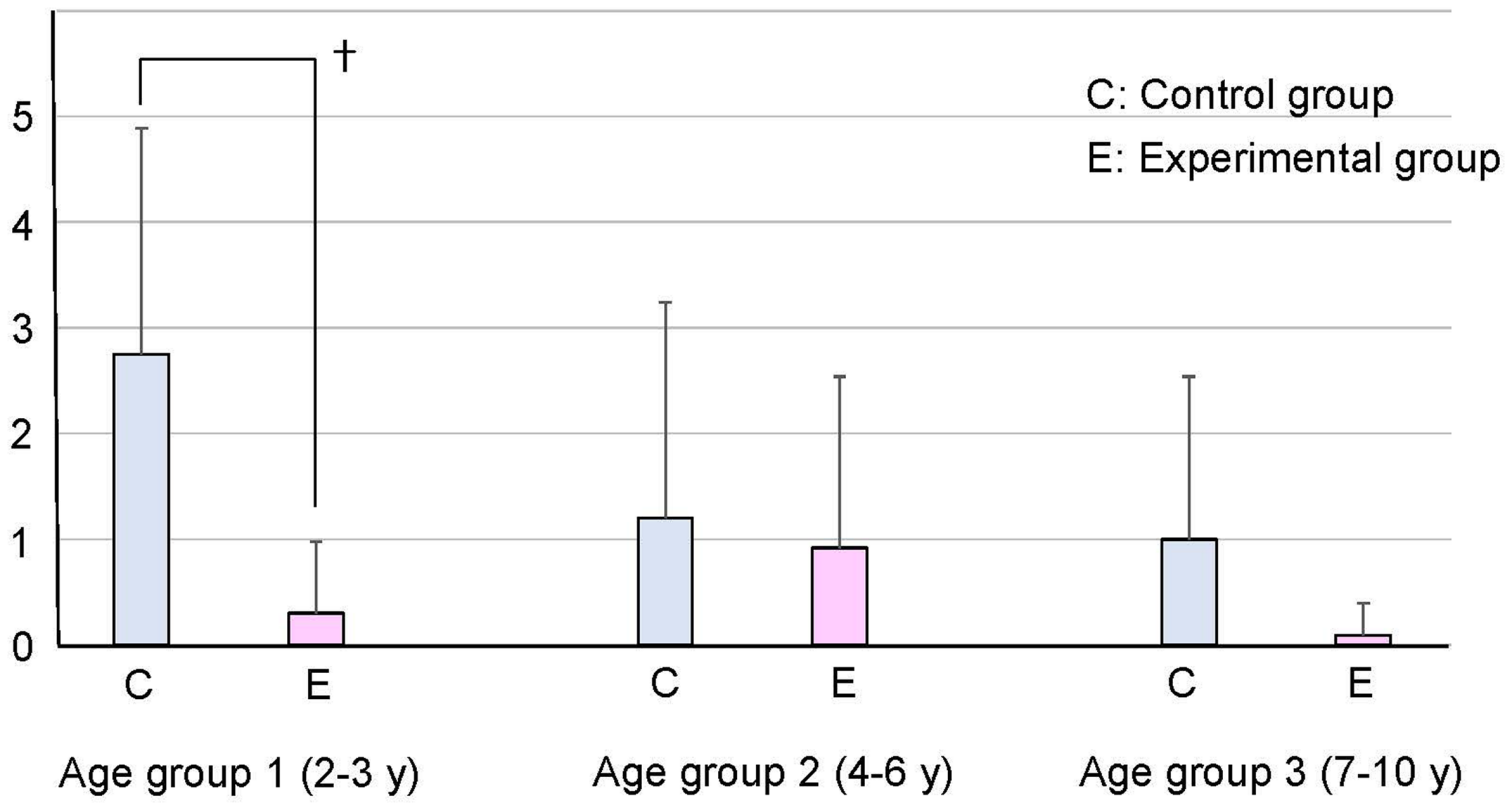
Behavioral score by age group The behavioral score was significantly lower in patients wearing a scented mask than those wearing an unscented one in age group 1 (†: p < 0.05).

There was no statistically significant difference in baseline patient characteristics between the control and experimental groups (Table 2). The primary outcome, the mask acceptance score, is shown in Figure 2 and Table 3. The mask acceptance score was significantly lower in the experimental than in the control group in patient group 1 (*p* < 0.05). Similarly, the behavioral score was significantly lower in the experimental than in the control group in group 1 (*p* < 0.05, Figure 3).

**Table 2:** Patient characteristics.

	Control group	Experimental group
Group 1 (age 2–3 y)	*n* = 12	*n* = 10
Age (months)	34 (6)	37 (7)
Height (cm)	89.0 (6.9)	91.3
Body weight (kg)	13.4 (2.9)	13.5 (1.7)
Gender, male: female	7: 5	6: 4
Type of surgery		
Inguinal and pubic surgery	6	3
Eye surgery	0	1
Ear or nose surgery	3	0
Skin surgery	3	1
Tonsillectomy	0	3
Others	0	2
Pulse rate in ward (bpm)	99 (11)	97 (14)
Group 2 (age 4–6 y)	*n* = 10	*n* = 12
Age (months)	65 (12)	67 (12)
Height (cm)	110.7 (8.6)	111.6
Body weight (kg)	18.2 (3.3)	19.1 (3.3)
Gender, male: female	4: 6	7: 5
Type of Surgery		
Inguinal and Pubic surgery	1	3
Eye surgery	3	2
Ear or Nose surgery	1	2
Skin surgery	4	3
Tonsillectomy	1	1
Others	0	1
Pulse rate in ward (bpm)	83 (13)	89 (18)
Group 3 (age 7–10 y)	*n* = 12	*n* = 11
Age (months)	104 (18)	105 (12)
Height (cm)	128.8 (7.5)	131.2
Body weight (kg)	27.8 (3.1)	29.6 (5.7)
Gender, male: female	7: 5	6: 5
Type of surgery		
Inguinal and pubic surgery	4	2
Eye surgery	2	4
Ear or nose surgery	3	1
Skin surgery	0	1
Tonsillectomy	2	2
Others	1	1
Pulse rate in ward (bpm)	81 (12)	88 (10)

bpm, beats per minute. Data are presented as number or mean (SD).

**Table 3:** Mask acceptance and behavioral scores.

	Control group	Experimental group
Group 1 (age 2–3 y)		
Mask acceptance score	3 (2–4)	1 (1–1)^a^
Behavioral score	2.8 (2.1)	0.3 (0.7)^a^
Group 2 (age 4–6 y)		
Mask acceptance score	1 (1–3.5)	1 (1–2.25)
Behavioral score	1.2 (2.0)	0.9 (1.6)
Group 3 (age 7–10 y)		
Mask acceptance score	1 (1–3)	1 (1–1)
Behavioral score	1.0 (1.5)	0.1 (0.3)

a *p* < 0.05 compared with the control group.

Mask acceptance scores are presented as median (IQR) and behavioral scores are presented as mean (SD).

**Figure 4:**
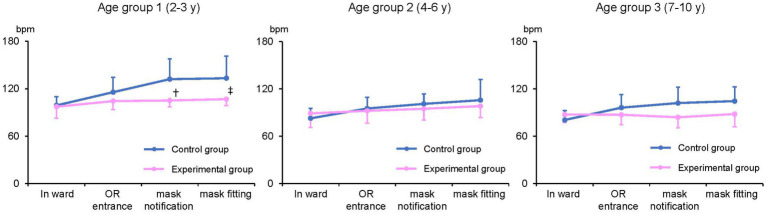
Pulse rate (PR) measurement by age group PR at mask notification and after mask fitting were significantly lower in patients wearing a scented mask than those wearing an unscented one in age group 1 (†, ‡: *p* < 0.01). bpm: beats per minute.

The changes in PR in each age group are shown in Figure 4. Mean PR in the ward did not differ between the groups in all age categories. In group 1, PRs were significantly lower in the experimental than in the control group at mask notification (105 ± 8 vs. 132 ± 26, *p* < 0.01) and just after mask fitting (105 ± 8 vs. 133 ± 28 vs.107 ± 8, p < 0.01), but not at the other time points. No significant difference in PR was observed between the control and experimental groups in patient groups 2 and 3.

## Discussion

This study evaluated mask acceptance scores in response to fitting with a scented or unscented face mask in pediatric patients categorized by age. The results of this study showed the use of a scented mask significantly improved mask acceptance at mask fitting and decreased PR in patients aged 2–3 years. These results might suggest that the use of a scented mask could attenuate stress responses to mask fitting in these younger patients.

Various approaches to facilitate slow induction have been examined in pediatric patients. Showing a children’s audiovisual program, selected by the patient or parent in advance, during slow induction was found to facilitate induction in patients aged 7–10 years (9). Patients aged 2–3 years had difficulty paying attention to videos before and during induction, indicating that video-assisted induction was not useful for these patients. In the present study, patients or parents chose a scented mask based on scent preference on the day before surgery. An analysis of olfactory capacity in healthy preverbal children showed that, for 98% of 105 children, the first presentation of the olfactory stimulus resulted in a modification of respiratory rhythm, a fixed gaze, and a decrease in mobility (12). In addition, 75% of the children aged ≥1 year held the olfactory stimulus (scented tissue) to the nose for more than 20 s, which may explain why patients aged 2–3 years in the present study showed good mask acceptance. Improvement in mask acceptance might enhance the success of slow induction in pediatric patients.

Mask acceptance, behavioral score, and PR in patients aged 4–6 years did not differ significantly between the experimental and control groups. Social perceptiveness is thought to develop from ages 4 to 6 years (13), suggesting that patients in this age group, even those fitted with unscented masks, might easily adapt to situations without fear. Use of the Induction Compliance Checklist (ICC) (14) to determine the quality of induction and mYPAS score to measure anxiety found no difference between placebo and flavored masks in patients aged 4–12 years (15). Although the number of highly anxious children differed in the placebo and flavor mask groups, the quality of induction was similar in patients aged 4–12 years. Similarly, the results of the present study showed no difference between the use of scented and unscented masks in children aged 4–10 years.

By contrast, the use of essential oil was found to promote the induction of anesthesia in children aged 5–14 years (2). In that study, children undergoing tooth extraction were randomly allocated to slow inductions with or without sweet orange essential oil. Children inhaling sweet orange essential oil were significantly more relaxed and cooperative during induction (72% vs. 23%, *p* < 0.05) and stated that they would prefer a similar anesthetic technique in the future (82% vs. 55%, *p* < 0.05). We expected mask acceptance would improve in the similar age group in our study because the patients had chosen a scented mask by themselves; however, there was no difference. The variety or intensity of the scents might affect the results of the study.

Studies of olfaction in infants have shown that newborns can distinguish the odor of their mother’s breast milk, which is important for feeding and bonding (16). Sense of smell is also important for protection from danger. However, evaluation of olfaction can be difficult among preverbal patients, and there is not much data for this age group. Children older than approximately 4 years of age can verbalize their experiences and score olfactory stimuli (13). Further research on olfaction is warranted for patients who are preverbal or unable to express themselves using words.

## Limitations

First, this study was performed in the setting with the presence of a parent for induction. Because pediatric patients pay attention to the words and behavior of their parents, the presence of a parent might have affected the study results. However, the presence of a parent or parents during the induction is generally accepted in the practice of pediatric anesthesia, hence the results of our study would rather reflect real-world data. Second, the attending anesthesiologists who put on the mask varied from resident to supervisor, indicating skills for pediatric anesthesia could vary among the anesthesiologists; this might have influenced the results of this study. Furthermore, we did not score the patient’s anxiety in the ward; it may be difficult to evaluate stress levels accurately by the mask acceptance score and the change in PR alone. Future studies should include measurements of mYPAS to determine anxiety. Mask acceptance is indeed the first gate for so-called slow induction; however, good acceptance may not always guarantee successful induction.

## Conclusion

The use of a scented face mask could improve mask acceptance before anesthesia induction in pediatric patients aged 2–3 years with the presence of a parent. The scented mask may facilitate the induction of general anesthesia in pediatric patients, although age may affect the effectiveness of the scented mask.

## Data availability statement

The raw data supporting the conclusions of this article will be made available by the authors, without undue reservation.

## Ethics statement

The studies involving human participants were reviewed and approved by The Jikei University Hospital Ethics Committee. Written informed consent to participate in this study was provided by the participants' legal guardian/next of kin.

## Author contributions

YA and KT conceived the idea of the study and substantially contributed to the study conceptualization. KT drafted the original manuscript. YA made substantial contributions to the study concept or the data analysis or interpretation. KT, YS, YH, EH, and AK contributed to the acquisition. NK supervised the conduct of this study and revised the manuscript critically for important intellectual content. TT advised the statistical analysis plan and statistical analyses and contributed to the interpretation of the results. All authors contributed to the article and approved the submitted version.

## Conflict of interest

The authors declare that the research was conducted in the absence of any commercial or financial relationships that could be construed as a potential conflict of interest.

## Publisher’s note

All claims expressed in this article are solely those of the authors and do not necessarily represent those of their affiliated organizations, or those of the publisher, the editors and the reviewers. Any product that may be evaluated in this article, or claim that may be made by its manufacturer, is not guaranteed or endorsed by the publisher.

## Footnotes

^1^https://upload.umin.ac.jp/cgi-open-bin/ctr_e/ctr_view.cgi?recptno=R000040819

## References

[1] Yamashita M Motokawa K. “Fruit-flavored” mask induction for children. Anesthesiology. (1986) 64:837. doi: 10.1097/00000542-198606000-00041, PMID: 3717659

[2] Mehta S Stone DN Whitehead HF. Use of essential oil to promote induction of anaesthesia in children. Anaesthesia. (1998) 53:720–1. doi: 10.1046/j.1365-2044.1998.537q-az0584q.x, PMID: 9771197

[3] Bromfalk Å Myrberg T Walldén J Engström Å Hultin M. Preoperative anxiety in preschool children: a randomized clinical trial comparing midazolam, clonidine, and dexmedetomidine. Paediatr Anaesth. (2021) 31:1225–33. doi: 10.1111/pan.14279, PMID: 34403548

[4] Kain ZN Mayes LC Wang SM Caramico LA Hofstadter MB. Parental presence during induction of anesthesia versus sedative premedication: which intervention is more effective? Anesthesiology. (1998) 89:1147–56, discussion 9A-10A. doi: 10.1097/00000542-199811000-00015, PMID: 9822003

[5] Gil Mayo D Sanabria Carretero P Gajate Martin L Alonso Calderón J Hernández Oliveros F Gomez Rojo M. Parental presence during induction of anesthesia improves compliance of the child and reduces emergence delirium. Eur J Pediatr Surg. (2021) 32:346–51. doi: 10.1055/s-0041-1732321, PMID: 34243210

[6] Golden L Pagala M Sukhavasi S Nagpal D Ahmad A Mahanta A. Giving toys to children reduces their anxiety about receiving premedication for surgery. Anesth Analg. (2006) 102:1070–2. doi: 10.1213/01.ane.0000198332.51475.50, PMID: 16551900

[7] Patel A Schieble T Davidson M Tran MC Schoenberg C Delphin E. Distraction with a hand-held video game reduces pediatric preoperative anxiety. Paediatr Anaesth. (2006) 16:1019–27. doi: 10.1111/j.1460-9592.2006.01914.x, PMID: 16972829

[8] Vagnoli L Caprilli S Robiglio A Messeri A. Clown doctors as a treatment for preoperative anxiety in children: a randomized, prospective study. Pediatrics. (2005) 116:e563–7. doi: 10.1542/peds.2005-0466, PMID: 16199685

[9] Abukawa Y Hiroki K Ozaki M. The change in pulse rate and behavioral score by using video assisted induction of pediatric anesthesia. Open J Anesthesiol. (2016) 6:45–50. doi: 10.4236/ojanes.2016.63007

[10] Wang L Huang L Zhang T Peng W. Comparison of intranasal dexmedetomidine and oral midazolam for premedication in pediatric dental patients under general anesthesia: a randomised clinical trial. Biomed Res Int. (2020) 2020:1–7. doi: 10.1155/2020/5142913 PMC7196136 32382556

[11] Chorney JM Kain ZN. Behavioral analysis of children’s response to induction of anesthesia. Anesth Analg. (2009) 109:1434–40. doi: 10.1213/ane.0b013e3181b412cf, PMID: 19713262

[12] Pomares CG Schirrer J Abadie V. Analysis of the olfactory capacity of healthy children before language acquisition. J Dev Behav Pediatr. (2002) 23:203–7. doi: 10.1097/00004703-200208000-00002, PMID: 12177565

[13] Schriever VA Zscheile L Gellrich J. Odor identification performance in children aged 3–6 years. Ped Res. (2021) 89:1304–9. doi: 10.1038/s41390-020-1083-3 PMC8370871 32712626

[14] Kain ZN MacLaren J McClain BC Saadat H Wang SM Mayes LC. Effects of age and emotionality on the effectiveness of midazolam administered preoperatively to children. Anesthesiology. (2007) 107:545–52. doi: 10.1097/01.anes.0000281895.81168.c3, PMID: 17893449

[15] Gupta A Mathew PJ Bhardwaj N. Flavored anesthetic masks for inhalational induction in children. Indian J Pediatr. (2017) 84:739–44. doi: 10.1007/s12098-017-2368-3, PMID: 28523393

[16] Varendi H Porter RH. Breast odour as the only maternal stimulus elicits crawling towards the odour source. Acta Paediatr. (2001) 90:372–5. doi: 10.1080/080352501750126131, PMID: 11332925

